# Lens Oscillations in the Human Eye. Implications for Post-Saccadic Suppression of Vision

**DOI:** 10.1371/journal.pone.0095764

**Published:** 2014-04-22

**Authors:** Juan Tabernero, Pablo Artal

**Affiliations:** Laboratorio de Óptica, Instituto Universitario de investigación en Óptica y Nanofísica, Universidad de Murcia, Murcia, Spain; Barrow Neurological Institute, United States of America

## Abstract

The eye changes gaze continuously from one visual stimulus to another. Using a high speed camera to record eye and lens movements we demonstrate how the crystalline lens sustains an inertial oscillatory decay movement immediately after every change of gaze. This behavior fit precisely with the movement of a classical damped harmonic oscillator. The time course of the oscillations range from 50 to 60 msec with an oscillation frequency of around 20 Hz. That has dramatic implications on the image quality at the retina on the very short times (∼50 msec) that follow the movement. However, it is well known that our vision is nearly suppressed on those periods (post-saccadic suppression). Both phenomenon follow similar time courses and therefore might be synchronized to avoid the visual impairment.

## Introduction

Oscillations of the crystalline lens after saccadic eye movements have been rarely described in the literature. Only when they are visually apparent by the naked eye of an external observer they are assessed under slit lamp examination (the whole lens trembles upon movements of the eye) and are known as "phakodonesis" [1]. If that phenomenon occurs in a pseudophakic eye, it is the intraocular lens (IOL) implanted during cataract surgery which wobbles and then it is called "pseudophakodonesis" [2]. In this last situation, the reason why IOL trembles might be intrinsic to the surgical procedure or even arise from a pathological weakness of the ciliary body. A similar phenomenon occurs when the iris trembles after eye movements. In this case, the associated pathology is called "iridodonesis" and it is the result of the inertial oscillations of the fluids in the anterior segment of the eye [2]–[3]. Any optical tracking technique using the pupil center location for the precise measurement of eye position can suffer post-saccadic artifacts ("ringing") due to this phenomenon [4]–[5].

Concerning lens wobbling, Dual Purkinje Image eye trackers [6] provide a better description of the phenomenon. These devices estimate direction of gaze using a calibrated relationship between eye movements and the relative change in the distance between the fourth Purkinje image (PIV; reflection from the back surface of the crystalline lens) and the first Purkinje image (PI; corneal front surface reflection). Tremulousness of the lens (induced by a saccadic eye movement) could also be recorded as the result of a quick oscillation of the PIV reflection with respect to the already stationary PI. Some authors have used these devices as an indirect method to measure and study lens wobbling after saccadic movements and to assess changes of the oscillations with the accommodation state of the lens [7], [8]. However, commercial eye trackers have some intrinsic inconveniences for the specific recording of lens wobbling in terms of visualization, extraction of data, tracking and customization of the illumination scheme and even temporal resolution of the camera sensors. For those reasons, in our lab and based on a previous developed instrument to measure lens position and misalignment [9], we have built a new experimental device to specifically observe and quantify human lens wobbling at high spatial and temporal resolution. In this work we systematically characterize the new method and provide clear and robust evidences of the lens oscillations after saccadic movements in young healthy subjects. More specifically, the dynamic of the lens wobbling is described in detail and optical simulations are performed to show the disturbing effects of lens wobbling in the retinal image.

## Materials and Methods

### Instrumentation

**Figure 1:**
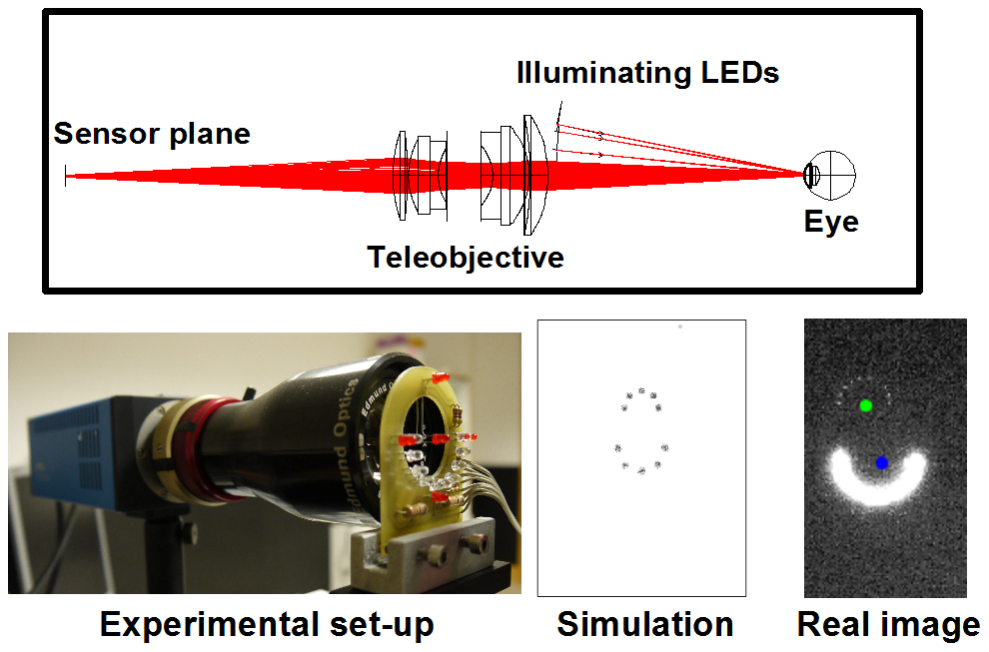
Overview of the experimental set-up and ray tracing simulation scheme. Upper panel: An eye model reflects light rays from a semicircular arrangement of point sources (simulating the array of illuminating LEDs). The reflected images from the point sources are captured through a teleobjective (working distance is 11 cm). The procedure to simulate the image formation of the corneal and posterior lens specular reflection images (PI and PIV images) consists of two sequential steps where the corresponding surfaces of the model eye are converted to mirrors. Lower panel, left: Picture of the actual set up at the lab with its main components: Camera, teleobjective, illumination and fixation LEDs. White LEDs (emitting IR light) in the image form the illumination semicircular source. Red (visible) fixation LEDs are used to generate the saccade movement of the subject by flickering from the center to a peripheral position alternatively (flickering frequency in this experiment was 1 Hz). Lower panels, right: Comparing the outcome of the computer model (left) to a real frame picture. This particular position corresponds to a 9 degrees upwards stationary position. Notice that PIV is optically inverted with respect to PI (negative magnification) and it is generally smaller than PI. The use of a semicircular source of light permits a very simple identification of each reflection.

The wobbling effect could be quantified recording the ocular reflections originated from a certain source of light (Purkinje images). With a previous version of our Purkinje-meter prototype [9], we were able to analyze the stationary position of the lens (or intraocular lens; IOL), and therefore to study the long term stability of different IOL designs [10]. The new device (a Dynamic Purkinje-meter) has been re-designed with significant modifications. Purkinje images were generated as in the previous device with a semicircular ring of Infrared LEDs, attached to a 0.5× telecentric teleobjective with working distance of 11 cm (Edmund Optics, York, UK), but in this case we used a CMOS camera sensor (pco.1200 hs, PCO AG, Kelheim, Germany) to capture a time sequence at high speed (400 frames per second) during saccadic eye movements (9° amplitude). The resolution of the sensor was 1280×1024 pixels and the length of the pixel was 12 µm (pixels were squares). With the telecentric objective attached to the camera and focused at 11 cm, one pixel length was equivalent to 24.5 µm on the image captured. The 9° saccades were induced by two flickering fixational red LEDs placed in a central and a peripheral position with respect to the objective-camera axis. Flickering frequency could be switched to 1, 0.5 or 0.33 Hz. In order to investigate the dependence of stability on the direction of the eye movement, we included four possible orientations for the flickering LEDs (nasal, temporal, inferior and superior). Figure 1 shows a ray tracing diagram of the setup (upper panel) and an actual picture of the instrument (left bottom side).

Immediately after a change of gaze, if the lens wobbles, a quick oscillation of the position of Purkinje image PIV (from posterior lens surfaces) with respect to PI (corneal reflection) can be observed. To track those positions (PI and PIV) and the pupil center, we programmed automatic (off-line) image processing routines (Mathematica 8.0, Wolfram Research, Champaign, IL). Pupil tracking was the result of applying thresholding, binarization, and edge detection techniques to each image frame to finally calculate the center of mass of the resulting pupil profile. To detect the positions of PI and PIV, in a first (manual) step, we selected one frame of the recorded time sequence and then we defined two regions of interest (ROI) around each of the reflections (PI and PIV). These two ROI were used as kernels to perform a normalized correlation with the rest of frames of the selected time sequence. On each correlated image frame, the pixel with the maximum brightness value marked the corresponding positions of PI and PIV.

**Figure 2:**
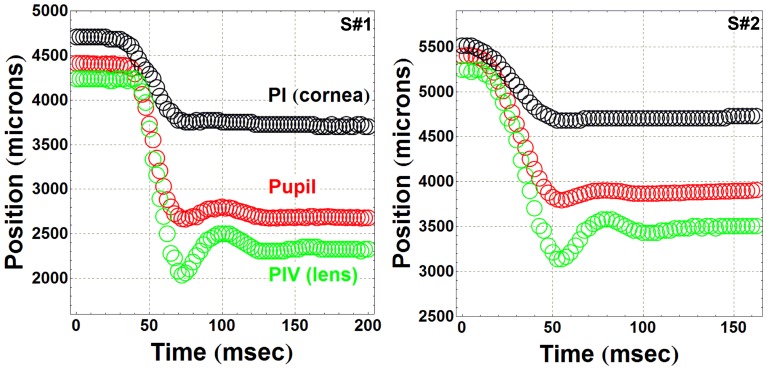
Tracking the pupil, PI (cornea) and PIV (lens). Data correspond to two different subjects performing a horizontal center-temporal 9° saccade. The reference position is the left bottom corner of the CMOS camera sensor. Black circles represents PI (corneal reflection) position as a function of time (no wobbling observed), red circles are pupil positions (oscillations are clearly observed) and green circles corresponds to PIV (lens reflection) where post-saccadic oscillations are visually evident.

Figure 2 shows examples of the tracking procedure for two different subjects. The horizontal coordinate of the position of PI, PIV and pupil center with respect to the lower left corner of the CMOS sensor were plotted as a function of time for a horizontal (center-temporal) 9° saccade movement. The corneal reflection (PI) remained stable after the saccade, marking a clear begin and end to the movement of the eye. However (PIV) showed a very characteristic oscillating movement immediately beginning after the eye globe was stabilized (PI did not move anymore). The pupil showed also some inertial oscillations (a non-pathological kind of iridodonesis). As an example of the experimental procedure, three processed video recordings (from three different subjects) were provided as supporting information movies (movie S1, movie S2 and movie S3). Each movie corresponded to a 9 degree saccade but with different orientations, center-downwards, center-upwards and center-temporal saccades. None of the subjects showed after-saccade PI (corneal) oscillations. Therefore, we assumed that PI could be used as a clear stable reference for measuring after-saccade lens oscillations.

Stability of the lens was assessed by fitting the relative movement of PIV with respect to PI to the solutions of a classical damped harmonic oscillator system:
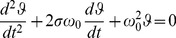
(1)where 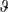 is the relative position of PIV with respect to PI; *t* represents time; σ is the damping ratio and ω_0_ is the undamped angular frequency of the movement. The fitted time sequence corresponded to the pure wobbling effect considering "time zero" as the time where the amplitude of wobbling is maximum (i.e. the distance from PI to PIV is maximum). This condition occurred immediately after the eye stops (The corneal reflection, PI, remained already stationary). As a result from the fitting procedure (a least square fitting routine performed also with Mathematica 8.0), we obtained a precise description of the lens wobbling in terms of the fitting parameters, i.e., the amplitude (the highest distance of PIV from PI with respect to the stationary distance), the oscillation frequency and the damping ratio. Additionally, we defined a time constant of the movement (stationary time) as the time that makes the oscillation amplitude decay to nearly its stationary value. We considered that point as the last time when the absolute value of the amplitude reached a 5% of the maximum peak.

### Subjects and procedure

Informed consent was obtained from all participants. The study protocol was in accordance with the guidelines of the Declaration of Helsinki and it was approved by the University of Murcia ethics committee. Eight subjects aged 28 to 42 (mean 35 years; standard deviation 5 years) took part in this experiment. No eye had a history of ocular surgery and all were nearly emmetropic (ie, 2.00 diopters or less of spherical equivalent). The measurements were taken with natural pupils (pharmacologically pupil dilation was not required). Lens wobbling was recorded in the right eye of each subject induced by vertical and horizontal saccades. Saccades were forced along the upper (9° saccade from central to upper position), lower (9° from central to down) and horizontal (9° from central to temporal) directions. The flickering frequency of the fixation LEDs was 1 Hz and the total time of the flickering was 15 secs, so that we recorded three different movies for each subject with a maximum of 7 center-upwards/downwards/temporal saccades.

## Results

**Figure 3:**
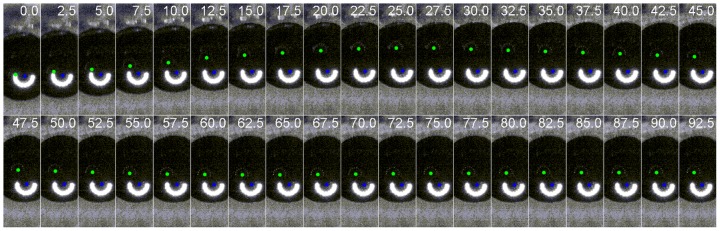
Time sequence of a 9° upwards saccade. Two semicircular images are seen on the pupil of the subject. They are the specular reflections of a semicircular infrared source. The brightest one, marked as a blue dot on its center, corresponds to the corneal reflection (first Purkinje image). The second one, marked with a green dot, is the fourth Purkinje image, a reflection from the back surface of the human lens, not as bright as the first one and optically inverted with respect to the first Purkinje. The relative movement of both images when the eye stops (in this particular case that happens at the 22.5 msec frame) corresponds to a movement of the lens in the stationary eye.

Based on the high speed recording of Purkinje images, we were able to obtain a precise description and characterization of the lens wobbling in terms of the amplitude (i.e. the largest distance of PIV from PI with respect to the stationary distance), the oscillation frequency, the damping ratio and the time course of the movement (stationary time). Figure 3 shows a typical sequence of images where lens wobbling is easily observed from the oscillation of PIV with respect to PI. Additionally, three fully processed video recordings (tracking of PI, PIV and pupil center) from three different subjects were provided as movie S1, movie S2 and movie S3 (showing upwards, downwards and temporal saccades).

**Figure 4:**
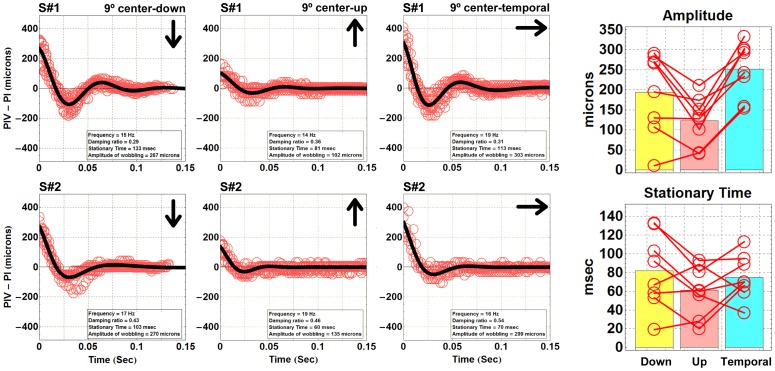
Wobbling data for two different subject (left panels) depending on the saccade orientation. Each plot displays the relative displacement of the fourth Purkinje image with respect to the corneal reflection as a function of time. The data (red open circles) corresponds to the wobbling effect recorded from all center-upwards, center-downwards and center-temporal saccades performed during the 14 seconds measurement, collected and overlapped on the same graph. The zero time corresponds to the maximum distance (maximum wobbling) between PI and PIV. The black solid lines correspond to fittings of the experimental data to the solutions of a damped harmonic oscillator model. Stationary time and wobbling amplitude (bar charts). Data corresponds to 8 young subjects who performed 9° center-upwards (pink), center-downwards (yellow) and center temporal (blue) saccadic movements. Bars represent the average values in each group and the empty red circles are the actual data. Red lines connect data obtained from the same subjects.

Figure 4 (six graphs on the left) shows data from two different subjects (S#1 and S#2). From each movie, we extracted all the wobbling data induced by the saccades during a 14 seconds sequence and we plotted them all in each graph (red open circles). All these data (PIV –PI versus time) were fitted to the solutions of the damped harmonic oscillator model (a sinusoidal exponential decay) from equation 1 (black solid line). In the figure, we presented data corresponding to upwards, downwards and temporal saccades of both subjects. For a better visual comparison, the sign of the PIV-PI displacement was normalized to be the same between all directions of movements. The accuracy of the fittings shows that a simple elastic model is adequate for the description of the phenomenon. Typical mean values of the fitting parameters that characterize the dynamic response of the lens for these particular 9° saccade movements are damping ratios from 0.3 to 0.7 (lens oscillations are under-damped), natural frequencies between 15 to 20 Hz, an average amplitude of 191 microns and an average stationary time of around 65 msec. Since the lens is joined to the ciliary muscle by a set of ligaments (the zonule), these parameters characterized the average dynamic response against eye movements of this physiological mechanism.

Interestingly, as shown in the bar charts of figure 4, when the subjects performed an upwards eye movement, the amplitude of the wobbling (123±60 µm) decreased with respect to downwards eye movements (194±103 µm). Also the stationary time increased when moving downwards (from 52±28 msec to 84±40 msec). Running with or against the gravity might have had an effect on the lens dynamic response to eye movements. It might be also that the distributions of the tensional forces of the zonular fibers around the lens were not fully symmetric [8]. However, when we analyzed the horizontal (center to temporal) saccades (abducting saccades), we obtained by far the largest values of wobbling amplitude (255±60 µm) along the three measured orientations. This effect was probably related to the peak velocities and to the forces generated by the oculo-motor system of the eye along the different gaze directions. It is known that abducting saccades generate faster peak velocities than adducting and vertical saccades [11]–[12]. Since lens wobbling is an inertial phenomenon, we expected more significant effects when the eye moved in a certain direction where stronger acceleration/deceleration forces were generated.

**Figure 5:**
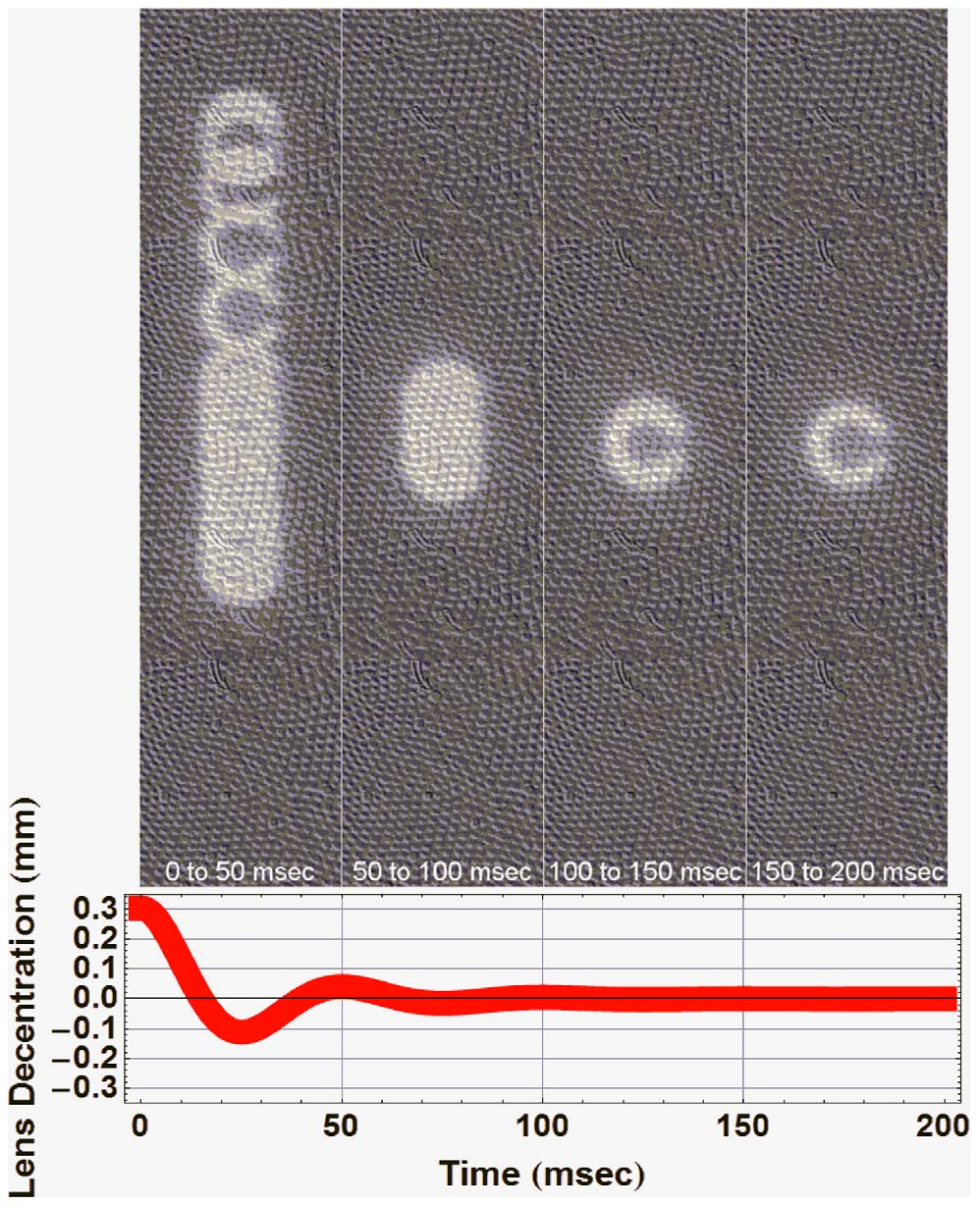
Example of a Landolt-C optotype wobbling over a retinal mosaic during different time intervals. The optotype oscillations are the optical consequence of a human lens wobbling simulated as an oscillating exponentially decay decentration of 0.3

We also performed a computer ray tracing simulation of the experimental procedure to provide a better description of the physical sources of lens wobbling. Figure 1 shows the ray tracing diagram of the set up (upper panel) together with a picture of the actual laboratory instrumentation (lower left panel). As a result from the ray-tracing simulation we were able to associate any change in the positions of the eye or the crystalline lens to a change in the relative positions of PI and PIV. The simulations of PI and PIV positions could also be compared to those of a real picture of PI and PIV (lower right). In particular, the image on the figure corresponded to a stationary frame when the real eye (and the model eye) was looking 9 degrees upwards. In the picture from the real eye, the vertical alignment of PI and PIV had some horizontal offset that was not present in the model eye. That offset was a consequence of some internal misalignment between lens and cornea that was not included in the simulation. Our computer model also allowed the simulation of the position and optical quality of any object imaged at the retinal plane by convolution of the object with the simulated Point Spread Function of the eye. In figure 5 we showed that a particular pattern of lens wobbling (induced as the temporal sequence of lens decentration shown on the lower panel) altered significantly the position of a Landolt C optotype at the retinal plane. We obtained a maximum image displacement of 0.3° for a 9° saccadic movement.

## Discussion

### Lens wobbling and post-saccadic suppression of vision

The lens wobbling produced a moving retinal image that would induce significant blur. This effect was represented in figure 5 for different time integrations after the saccadic movement and compared as a reference with the size of the foveal photoreceptors (an animated sequence of this figure was also included as movie S4). Despite the large blur, any potentially adverse visual effect would not be noticeable unless the time constant of lens wobbling (65 msec) greatly exceeds the time constant of the post-saccadic suppression time (which is in average around 50 msec). It is believed that this suppression has a neural origin [13], [14]. Simulated saccades performed with a movable mirror (keeping the eye stationary) produced little suppression when the targets were presented clearly over uniform backgrounds [14]. Therefore it has been suggested that the reason of saccadic vision impairment could not be generated only by an image being quickly displaced across the retina. However, that experiment did not include the oscillating effects on the retinal image produced by lens wobbling and it could not fully discard that the explanation of the post-saccadic suppression of vision had additionally an "optical" origin, especially when both post-saccadic suppression time and lens wobbling presented so similar time courses. It might even be that the mechanism of post saccadic suppression of vision was generated as a protective response against the retinal image blur generated not only by the saccade displacement [15] but also by the consequent lens wobbling effect.

### The physical sources and consequences of lens wobbling

Using ray tracing optical modeling [16], we estimated that 0.3 mm of lens decentration explained well an average lens wobbling amplitude of 200 microns displacement between PI and PIV. That amplitude could also be induced by lens tilt alone. However, only tilts starting from 4 degrees would generate such displacements. Given that our saccades had an amplitude of 9 degrees it seems unlikely that a 4 degree lens tilt (nearly half of the saccade amplitude) was responsible alone for the wobbling amplitude, although a combination of both parameters (small lens tilt and decentration) was certainly possible. Considering only a decentration amplitude of 0.3 mm as the source of lens wobbling, we also estimated the maximum retinal image displacement expected as the consequence of the lens oscillations. We obtained a maximum shift of 88 microns (visually, it represented a 0.3° angle) for a 9° saccade which is equivalent to 20 min arc in visual angle yielding an extremely poor post-saccadic visual acuity of 0.05 decimal (20/400). These are significant numbers that can have important effects on those experiments who include manipulation and location of stimulus immediately after a saccade [17]–[18].

### Lens wobbling and ocular diseases. Conclusions

Although we showed that lens wobbling can be easily recorded with full detail in normal healthy subjects, another important aspect of this work is the potential implication that those records might have as a reference for different pathological populations. As an example, it is expected that subjects with pseudoexfoliation syndrome (a disease that can alter the zonule fibers structure and consequently might release some capsular tension) had a modified lens dynamic response compared to normal healthy subjects. Another example might be those subjects with Marfan syndrome (very often characterized by a dislocated crystalline lens). The methodology here proposed can even be applied to subjects implanted with different intraocular lenses to study the stability of new lenses and its different models of haptics or stabilizers, like capsular tension rings. In general, it can be potentially used to improve diagnosis and monitor any disease that affects stability of the crystalline lens or any intraocular lens.

In conclusion, we have demonstrated that the human lens wobbles as a classical damped harmonic oscillator where upwards, downwards and temporal saccadic movements induced different dynamics. Interestingly, we reported a temporal parallelism between lens wobbling and the phenomenon of post saccadic suppression of vision. With the new designed instrumentation, further experiments to test new hypothesis are now certainly possible as well as the study and improved diagnosis of some diseases that affect the dynamic stability of the crystalline lens and its zonular support.

## Supporting Information

Movie S1 **Movie S1 corresponded to a 9° center to temporal (abducting) saccade.** The tracking of the 1st Purkinje image (corneal reflection) was marked with a white filled circle while tracking of the 4th Purkinje image (lens posterior surface reflection) was marked in green. The pupil profile was characterized with a solid red line and its center marked in a red dot. Crystalline lens reflection (4th Purkinje image) wobbled for a fraction of a second after the saccadic movement. Some wobbling of the pupil center was also visible but to a less extent than the crystalline lens wobbling.
(GIF)

Movie S2 **Movie S2 corresponded to a 9° center-down saccade.** Details were identical to movie S1.
(GIF)

Movie S3 **Movie S3 corresponded to a 9° center-up saccade.** Details were identical to movie S1.
(GIF)

Movie S4 **A Landolt C optotype wobbling over a retinal mosaic.** The Landolt C wobbling was the optical consequence of the oscillations of the human lens. They were simulated as a lens decentration that changes as an oscillating exponential decay of 0.3 mm of maximum amplitude. The maximum shift in the position of the optotype over the retinal mosaic was 88 microns.
(GIF)

## References

[1] Bartholomew RS (1970) Phakodonesis. A sign of incipient lens displacement. Brit J Ophthal 54: 663–666. 5472193 10.1136/bjo.54.10.663 PMC1208016

[2] Jacobi KW, Jagger WS (1981) Physical forces involved in pseudophacodonesis and iridodonesis. Graefes Arch Clin Exp Ophthalmol 216: 49–53. 10.1007/BF00407776 6909024

[3] Miller D, Doane MG (1984) High-speed photographic evaluation of intraocular lens movements. Am J Ophthalmol 97: 752–9. 6731539 10.1016/0002-9394(84)90508-7

[4] Kimmel DL, Mammo D, Newsome WT (2012) Tracking the eye non-invasively: simultaneous comparison of the scleral search coil and optical tracking techniques in the macaque monkey. Front. Behav. Neurosci 6: 49. 22912608 10.3389/fnbeh.2012.00049 PMC3418577

[5] Nyström M, Hooge I, Holmqvist K (2013) Post-saccadic oscillations in eye movement data recorded with pupil-based eye trackers reflect motion of the pupil inside the iris. Vision Res 92: 59–66. 24096093 10.1016/j.visres.2013.09.009

[6] Crane HD, Steele CM (1985) Generation-V dual-Purkinje-image eyetracker. Appl Optics 24: 527–537. 10.1364/ao.24.000527 18216982

[7] Deubel H, Bridgeman B (1995) Fourth purkinje image signals reveal eye-lens deviations and retinal image distortions during saccades. Vision Res 35: 529–538. 7900293 10.1016/0042-6989(94)00146-d

[8] He L, Donnelly III WJ, Stevenson SB, Glasser A (2010) Saccadic lens instability increases with accommodative stimulus in presbyopes. J Vis 10: 1–16. 10.1167/10.4.14 PMC2913422 20465334

[9] Tabernero J, Benito A, Nourrit V, Artal P (2006) Instrument for measuring the misalignments of ocular surfaces. Opt Express 14: 10945–10956. 19529508 10.1364/oe.14.010945

[10] Crnej A, Hirnschall N, Nishi Y, Gangwani V, Tabernero J, et al (2011) Impact of intraocular lens haptic design and orientation on decentration and tilt. J Cataract Refract Surg 37: 1768–1774. 21840681 10.1016/j.jcrs.2011.04.028

[11] Collewijn H, Erkelens CJ, Steinman RM (1988) Binocular co-ordination of human horizontal saccadic eye movements. J Physiol 404: 157–182. 3253429 10.1113/jphysiol.1988.sp017284 PMC1190820

[12] Collewijn H, Erkelens CJ, Steinman RM (1988) Binocular co-ordination of human vertical saccadic eye movements. J Physiol 404: 183–197. 3253430 10.1113/jphysiol.1988.sp017285 PMC1190821

[13] Burr DC, Morrone MC, Ross J (1994) Selective suppression of the magnocellular visual pathway during saccadic eye movements. Nature 371: 511–513. 7935763 10.1038/371511a0

[14] Diamond MR, Ross J, Morrone MC (2000) Extraretinal control of saccadic suppression. J Neurosci 20: 3449–3455. 10777808 10.1523/JNEUROSCI.20-09-03449.2000 PMC6773104

[15] MacKay D (1970) Elevation of visual threshold by displacement of retinal image. Nature 225: 90–92. 5410213 10.1038/225090a0

[16] Tabernero J, Piers P, Benito A, Redondo M, Artal P (2006) Predicting the optical performance of eyes implanted with IOLs to correct spherical aberration. Invest Ophthalmol Vis Sci 47: 4651–4658. 17003464 10.1167/iovs.06-0444

[17] Deubel H, Schneider WX, Bridgeman B (1995) Perceptual consequences of ocular lens overshoot during saccadic eye movements. Vision Res 35: 2987–2902. 10.1016/0042-6989(95)00042-x 8533329

[18] Deubel H, Bridgeman B (1996) Postsaccadic target blanking prevents saccadic suppression of image displacement. Vision Res 36: 985–996. 8736258 10.1016/0042-6989(95)00203-0

