# Supplementary figures and images for: Lens Oscillations in the Human Eye. Implications for Post-Saccadic Suppression of Vision
Source: PLoS One. 2014 Apr 22;9(4):e95764. doi: 10.1371/journal.pone.0095764 (PMC3995773; doi:10.1371/journal.pone.0095764)

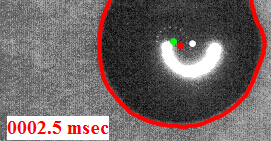

Supplement: Movie S1 — corresponded to a 9° center to temporal (abducting) saccade. The tracking of the 1st Purkinje image (corneal reflection) was marked with a white filled circle while tracking of the 4th Purkinje image (lens posterior surface reflection) was marked in green. The pupil profile was characterized with a solid red line and its center marked in a red dot. Crystalline lens reflection (4th Purkinje image) wobbled for a fraction of a second after the saccadic movement. Some wobbling of the pupil center was also visible but to a less extent than the crystalline lens wobbling. (GIF) [file pone.0095764.s001.gif]

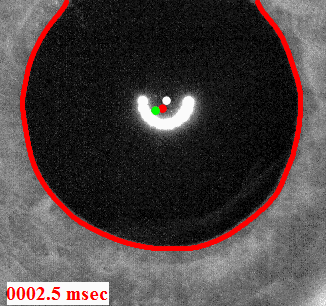

Supplement: Movie S2 — corresponded to a 9° center-down saccade. Details were identical to movie S1. (GIF) [file pone.0095764.s002.gif]

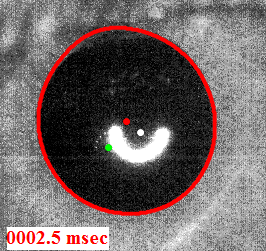

Supplement: Movie S3 — corresponded to a 9° center-up saccade. Details were identical to movie S1. (GIF) [file pone.0095764.s003.gif]

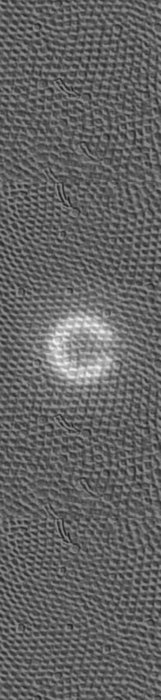

Supplement: Movie S4 — A Landolt C optotype wobbling over a retinal mosaic. The Landolt C wobbling was the optical consequence of the oscillations of the human lens. They were simulated as a lens decentration that changes as an oscillating exponential decay of 0.3 mm of maximum amplitude. The maximum shift in the position of the optotype over the retinal mosaic was 88 microns. (GIF) [file pone.0095764.s004.gif]
